# Obesity Outweighs Protection Conferred by Adjuvanted Influenza Vaccination

**DOI:** 10.1128/mBio.01144-16

**Published:** 2016-08-02

**Authors:** Erik A. Karlsson, Tomer Hertz, Cydney Johnson, Andrew Mehle, Florian Krammer, Stacey Schultz-Cherry

**Affiliations:** a Department of Infectious Diseases, St. Jude Children’s Research Hospital, Memphis, Tennessee, USA; b The Shraga Segal Department of Microbiology, Immunology and Genetics, Ben-Gurion University of the Negev, Be’er-Sheva, Israel; c National Institute of Biotechnology in the Negev, Be’er-Sheva, Israel; d Vaccine and Infectious Disease Division, Fred Hutchinson Cancer Research Center, Seattle, Washington, USA; e Department of Medical Microbiology and Immunology, University of Wisconsin—Madison, Madison, Wisconsin, USA; f Department of Microbiology, Icahn School of Medicine at Mount Sinai, New York, USA

## Abstract

Obesity is a risk factor for developing severe influenza virus infection, making vaccination of utmost importance for this high-risk population. However, vaccinated obese animals and adults have decreased neutralizing antibody responses. In these studies, we tested the hypothesis that the addition of either alum or a squalene-based adjuvant (AS03) to an influenza vaccine would improve neutralizing antibody responses and protect obese mice from challenge. Our studies demonstrate that adjuvanted vaccine does increase both neutralizing and nonneutralizing antibody levels compared to vaccine alone. Although obese mice mount significantly decreased virus-specific antibody responses, both the breadth and the magnitude of the responses against hemagglutinin (HA) and neuraminidase (NA) are decreased compared to the responses in lean mice. Importantly, even with a greater than fourfold increase in neutralizing antibody levels, obese mice are not protected against influenza virus challenge and viral loads remain elevated in the respiratory tract. Increasing the antigen dose affords no added protection, and a decreasing viral dose did not fully mitigate the increased mortality seen in obese mice. Overall, these studies highlight that, while the use of an adjuvant does improve seroconversion, vaccination does not fully protect obese mice from influenza virus challenge, possibly due to the increased sensitivity of obese animals to infection. Given the continued increase in the global obesity epidemic, our findings have important implications for public health.

## IMPORTANCE

Vaccination is the most effective strategy for preventing influenza virus infection and is a key component for pandemic preparedness. However, vaccines may fail to provide optimal protection in high-risk groups, including overweight and obese individuals. Given the worldwide obesity epidemic, it is imperative that we understand and improve vaccine efficacy. No work to date has investigated whether adjuvants increase the protective capacity of influenza vaccines in the obese host. In these studies, we show that adjuvants increased the neutralizing and nonneutralizing antibody responses during vaccination of lean and obese mice to levels considered “protective,” and yet, obese mice still succumbed to infection. This vulnerability is likely due to a combination of factors, including the increased susceptibility of obese animals to develop severe and even lethal disease when infected with very low viral titers. Our studies highlight the critical public health need to translate these findings and better understand vaccination in this increasing population.

## INTRODUCTION

The 2009 H1N1 pandemic provided the first evidence that obesity was a risk factor for developing influenza-related complications, including hospitalization and even increased mortality (1). This increased severity is not limited to the 2009 pandemic virus [A(pdmH1N1)]. Obesity is also linked to more severe disease with the avian A(H7N9) viruses, which first caused human infections in March 2013 (1–3). Given that we are currently facing our fourth wave of A(H7N9) human infections and that nearly 10% of the world’s adult population, as well as 42 million children under the age of 5 (4), are obese (body mass index [BMI] of >30 kg/m^2^), it is imperative that we understand the effectiveness of current influenza control and prevention strategies in this population.

Arguably, vaccination is the best prevention against influenza virus (5), and it is a key component for the preparedness and response to emerging influenza virus strains, including A(H7N9) viruses (6). Unfortunately, vaccines against avian influenza viruses have been poorly immunogenic in mammals, including humans, in spite of increased antigenic dose (7–9). Adjuvants are an effective means to increase humoral responses to influenza vaccines (10–15). Recent studies in ferrets (14) and humans (13, 15) demonstrated that administering vaccines with squalene oil-in-water adjuvants (MF59 and AS03) resulted in increased serological responses against a monovalent A(H7N9) influenza vaccine. However, no studies to date have examined the effectiveness of vaccines against emerging influenza viruses in the obese host, nor have they explored the effectiveness of adjuvants in this high-risk population. This is crucial, given that obesity has been associated with decreased response to seasonal influenza vaccines in animal models and human studies (16–18).

In these studies, we tested the efficacy of alum-adjuvanted, AS03-adjuvanted, or nonadjuvanted A(pdmH1N1) and A(H7N9) vaccines in lean and obese mice. Given the poor immunogenicity of seasonal vaccines in obese populations, we hypothesized that adjuvanted vaccine would improve humoral responses and protect obese mice from influenza virus challenge. While both types of adjuvanted vaccine did result in seroconversion and the generation of neutralizing and nonneutralizing antibodies, obese mice were not protected from challenge and had delayed viral clearance compared to lean mice. Increasing the antigenic dose had no impact on protection. Using several distinct methodologies, we found that the overall breadth and magnitude of the humoral response to both the viral hemagglutinin (HA) and neuraminidase (NA) were significantly decreased in the obese mice even with the addition of AS03. This result, combined with the increased disease severity in the face of low levels of virus, likely results in reduced protection from viral challenge, even when neutralizing antibodies levels reach reportedly protective titers (i.e., >1:40) (19). Given that in the United States alone, severe obesity has been forecasted to increase by 130% in the next two decades (20) and >70% of the European population is predicted to be overweight by 2030 (21), understanding why overweight/obesity results in impaired vaccine responses and increased susceptibility to severe infection are paramount for public health.

## RESULTS

### Adjuvanted vaccines increase seroconversion in obese mice.

**FIG 1:**
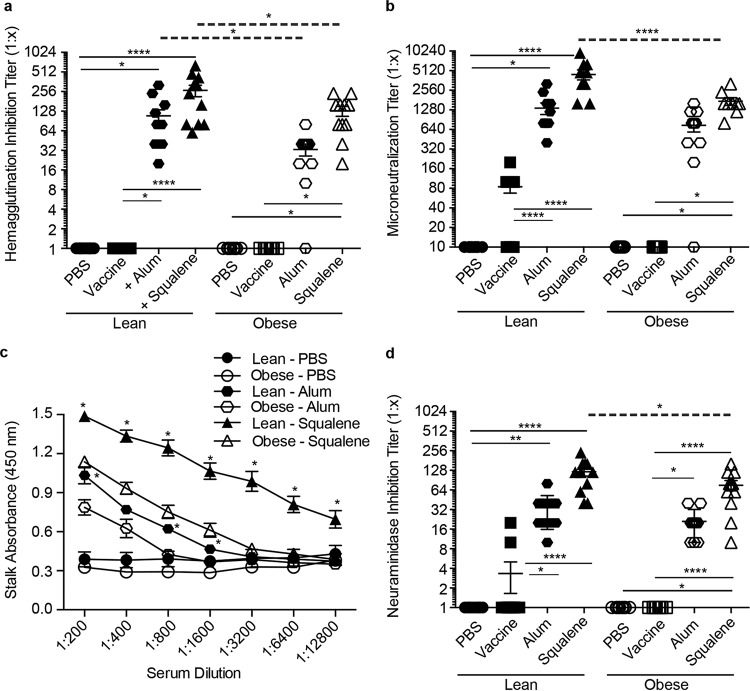
H7 postboost serology data. Eight groups of lean (solid symbols) or obese (open symbols) mice (*n* = 10 or 11/type/group) were vaccinated with PBS (circles), vaccine alone (squares), vaccine plus alum adjuvant (octahedrons), or vaccine plus squalene adjuvant (triangles). Three weeks postvaccination, mice were boosted, and serum was collected 3 weeks postboost. Postboost serum was analyzed for hemagglutination inhibition (HAI) (a), microneutralization (MN) (b), stalk antibody (c), and neuraminidase inhibition (NAI) (d) against influenza virus A/Anhui/1/2013 (H7N9). For HAI, MN, and NAI, data are presented as individual data points plus mean values ± standard errors. For stalk antibody, data are presented as mean absorbance values ± standard errors for mock-treated and vaccine-plus-adjuvant groups. Statistical significance was determined using ANOVA, with vaccine strategy and mouse type as the main effects. Tukey’s test was used for *post hoc* comparison. Differences were considered significant at a *P* value of <0.05. *, *P* < 0.05; **, *P* < 0.005; ***, *P* < 0.0005; ****, *P* < 0.00005. Solid lines indicate significance within vaccine strategies, and dashed lines indicate significance between lean and obese groups.

**FIG 2:**
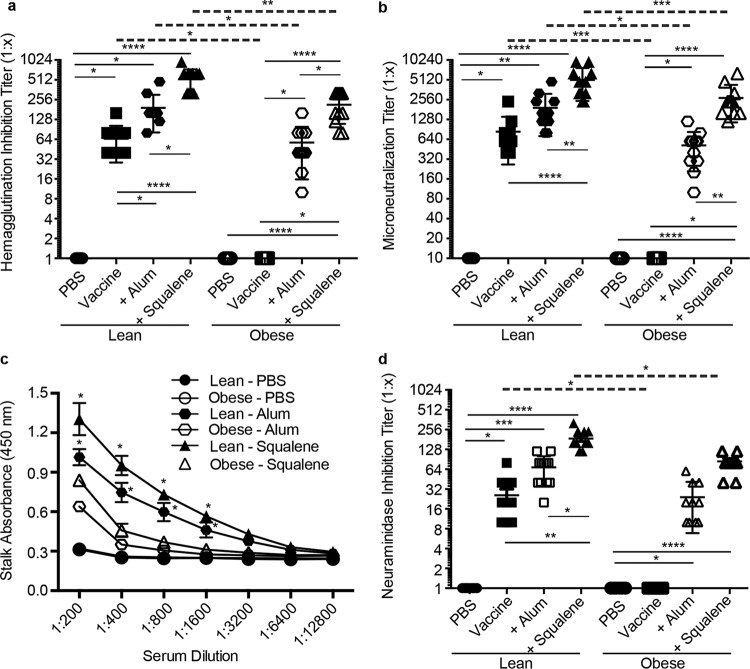
H1 postboost serology data. Eight groups of lean (solid symbols) or obese (open symbols) mice (*n* = 10 or 11/type/group) were vaccinated with PBS (circles), vaccine alone (squares), vaccine plus alum adjuvant (octahedrons), or vaccine plus squalene adjuvant (triangles). Three weeks postvaccination, mice were boosted, and serum was collected 3 weeks postboost. Postboost serum was analyzed for hemagglutination inhibition (HAI) (a), microneutralization (MN) (b), stalk antibody (c), and neuraminidase inhibition (NAI) (d) against influenza virus A/California/04/2009 (pdmH1N1). For HAI, MN, and NAI, data are presented as individual data points plus mean values ± standard errors. For stalk antibody, data are presented as mean absorbance values ± standard errors. Statistical significance was determined using ANOVA, with vaccine strategy and mouse type as the main effects. Tukey’s test was used for *post hoc* comparison. Differences were considered significant at a *P* value of <0.05. *, *P* < 0.05; **, *P* < 0.005; ***, *P* < 0.0005; ****, *P* < 0.00005. Solid lines indicate significance within vaccine strategies, and dashed lines indicate significance between lean and obese groups.

Previous studies have shown that obese mice mount poor serological responses to seasonal influenza vaccination (17, 18). To determine whether adjuvants improved these responses, C57BL/6 (wild type [WT] lean) and B6.Cg-*Lep^ob^*/J (obese) mice were vaccinated with the A(H7N9) vaccine manufactured by Sanofi-Pasteur or with seasonal vaccine against A/California/04/2009 (pdmH1N1) in the presence or absence of alum or AS03 adjuvant following the scheme in Fig. S1. Both alum and AS03 adjuvant increased neutralizing antibody titers as measured by hemagglutination inhibition (HAI) and microneutralization (MN) in both lean and obese mice compared to the titers with vaccine alone, although the titers were significantly lower when using alum than with AS03. In addition, the titers in the obese animals were significantly decreased compared to those in lean controls (Fig. 1 and 2A and B, respectively). With the A(H7N9) vaccine, the HAI titers ranged from 1:20 to 1:240 in obese mice receiving adjuvanted vaccine, while in lean mice, the HAI titers ranged from 1:80 to 1:620 (Fig. 1A). A similar trend was observed by measuring MN, where 100% of the lean mice receiving adjuvanted vaccine had MN titers ranging from 1:1,600 to 1:9,600, while the MN titers in obese mice were significantly decreased, ranging from 1:800 to 1:3,200 (Fig. 1B). In contrast to the obese mice, 80% of the lean mice receiving unadjuvanted vaccine also had increased MN titers, ranging from 1:100 to 1:200 (Fig. 1B). Similarly, the addition of adjuvant to seasonal A(pdmH1N1) vaccine also increased the HAI and MN titers in both lean and obese animals, with slightly decreased responses overall in obese mice compared to those in lean controls (Fig. 2A and B).

In addition to stimulating neutralizing antibodies toward the HA globular head domain, the generation of antibodies toward other domains of the virus, including the highly conserved stalk region or the NA protein, can also contribute to protective responses following vaccination (22, 23). To date, no studies have determined the impact of obesity on the generation of broadly neutralizing stalk antibodies or neuraminidase-inhibiting (NI) antibodies. Therefore, we measured H7 and H1 stalk and NI antibody levels in vaccinated mice by enzyme-linked immunosorbent assay (ELISA) and enzyme-linked lectin assay (ELLA), respectively. Virus-specific stalk antibody levels increased in both lean and obese mice even with vaccine alone (Fig. S2), and adjuvanted vaccination increased the stalk responses compared to those with vaccine alone; however, obese mice had significantly decreased responses with both adjuvants compared to their lean counterparts (Fig. 1C and 2C). Similarly, the addition of both adjuvants to the vaccine greatly increased NI antibodies in both the lean (NI range, 1:40 to 1:240) and obese (NI range, 1:20 to 1:160) mice, although the levels in obese mice were again significantly lower (Fig. 1D). Again, a very similar trend was observed with responses to the seasonal pdmH1N1 vaccine (Fig. 2D). Overall, these data indicate that, despite being slightly decreased compared to lean controls, serological responses to both mammalian and avian influenza vaccines are increased by adjuvanted vaccines in the obese host.

### Obesity affects the breadth and magnitude of the antibody response.

**FIG 3:**
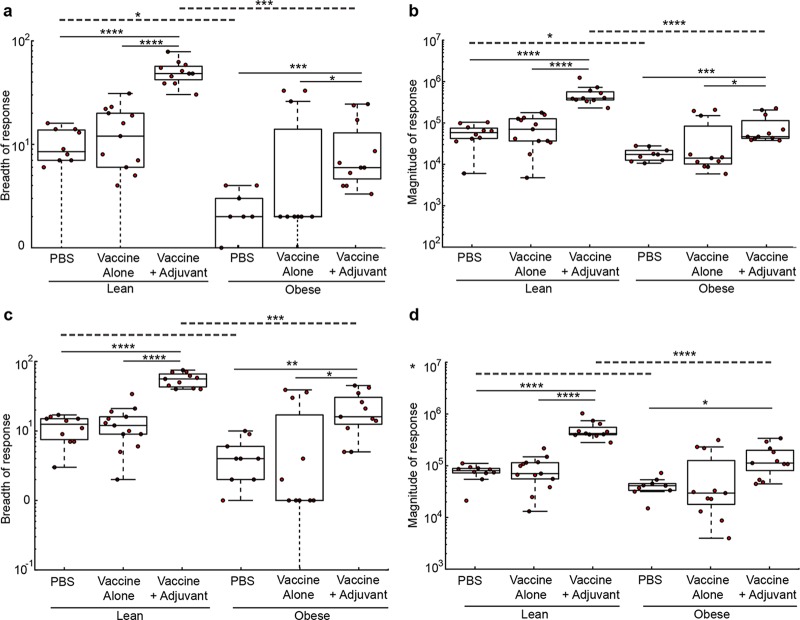
Obesity impacts the magnitude and breadth of antibody response to influenza vaccination. Antigen microarrays were used to examine the differences in the magnitude and breadth of antibodies specific for influenza virus A(H7N9) HA (a, b) or NA (c, d) protein between lean and obese groups. Data from individual lean and obese mice (red dots) from different vaccine strategies are presented as the breadth (a, c) or magnitude (b, d) of the overall response. The top and bottom edges of the boxplots represent the 1st and 3rd data quartiles, and whiskers denote 1.5 times the interquartile range (IQR). The median response of each group is represented as a horizontal line within each boxplot. Dots represent the actual responses of individual animals. Statistical comparisons were computed using the Wilcoxon rank sum test, and adjustments for multiplicity were computed using the Bonferroni correction. Differences were considered significant at a *P* value of <0.05. *, *P* < 0.05; **, *P* < 0.005; ***, *P* < 0.0005; ****, *P* < 0.00005. Solid lines indicate significance within vaccine strategies, and dashed lines indicate significance between lean and obese groups.

**FIG 4:**
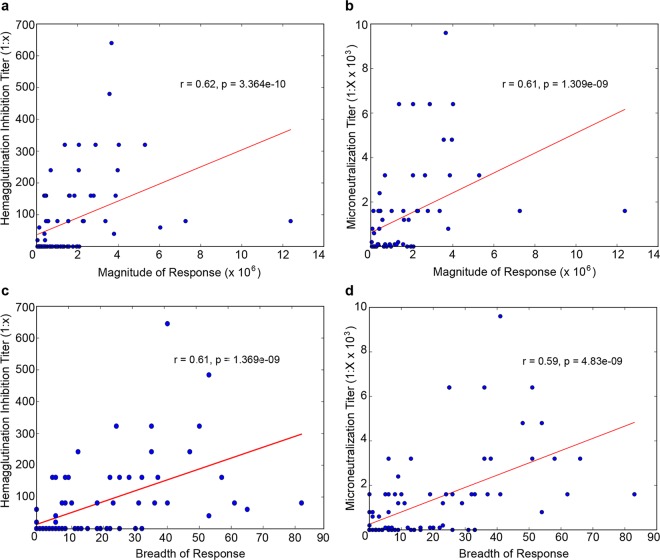
The magnitudes and breadth of responses directly correspond to the serological data. The data for magnitude (a, b) and breadth (c, d) of antibodies specific for influenza virus A(H7N9) HA, derived from antigen microarrays (AM), were correlated with hemagglutination inhibition (HAI) (a, c) and microneutralization (MN) (b, d) data from individual mice. Associations between AM data and HAI and MN assay results were analyzed for statistical significance using Spearman’s rank-order correlation.

Neutralizing and nonneutralizing antibody titers were reduced in the obese mice. However, the ELISA does not tell us whether obesity modulates the antibody repertoire by modifying the frequency of antibodies specific for particular HA or NA epitopes or whether there is a generalized decrease in response to all epitopes. To examine this and have a snapshot of the impact of obesity on the overall IgG response to vaccination, we evaluated the prevalence of antibodies specific for the A(H7N9) HA (Fig. 3A and B) or NA (Fig. 3C and D) proteins as described previously (24). Briefly, we generated antigen microarrays (AM), using 20-amino-acid peptides with an overlap of 15 amino acids, to provide dense coverage of potential A(H7N9) HA and NA epitopes. The sera from the different groups were incubated on the microarrays, and the amount of IgG bound to each epitope quantified by measuring the fluorescence intensity of antibodies bound to mouse IgG. To quantify these effects, we summarized the AM data using two commonly used summary statistics: overall response breadth (the number of epitopes targeted) and magnitude (amount of antibody). We then compared the magnitude and breadth of each of the six experimental groups in the study. In concurrence with the inhibition assays and ELISA/ELLA data, we found that vaccinated lean mice generated stronger and more broad antibody responses to the H7 HA (Fig. 3A and B) and N9 (Fig. 3C and D) peptide antigens than did obese mice. These differences in magnitude and breadth directly correlated with MN and HAI titers on an individual mouse basis (Fig. 4). Overall, these studies show that an adjuvanted vaccine increases the levels of both neutralizing and nonneutralizing antibodies in obese mice, although the breadth and the magnitude of the response are significantly lower than in lean mice.

### Despite seroconversion, obese mice are not protected during influenza virus challenge.

**FIG 5:**
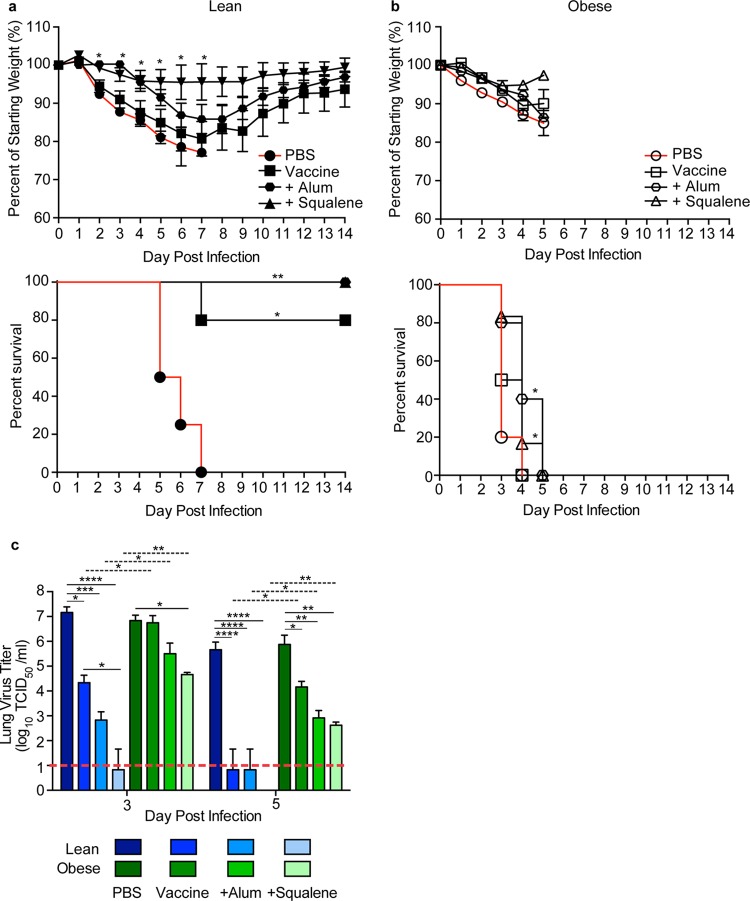
Survival, weight loss, and lung viral titers in vaccinated lean and obese mice following H7N9 virus challenge. (a, b) Three weeks postboost, lean (solid symbols) (a) and obese (open symbols) (b) mice (*n* = 5 or 6/type/group) were challenged with 100× MLD_50_ of influenza virus A/Anhui/1/2013. The mice were monitored for weight and survival daily for 14 days postinfection. Weight data are presented as mean values ± standard errors. Statistical significance was determined using ANOVA, with a *P* value of <0.05 deemed significant compared to the PBS controls. Survival data are presented as the percentages of animals surviving among the total number monitored. Statistical significance was determined by log rank (Mantel-Cox) test, with a *P* value of <0.05 deemed significant compared to the PBS control group. *, *P* < 0.05; **, *P* < 0.005. (c) Viral titers were determined in lungs from vaccinated lean (blue) and obese (green) mice at day 3 and day 5 postinfection with influenza virus A/Anhui/1/2013 (H7N9). Data are presented as mean log10 TCID_50_/ml ± standard error. Statistical significance was determined using ANOVA, with vaccine strategy, mouse type, and day postinfection as the main effects. Tukey’s test was used for *post hoc* comparison between days postinfection, mouse types, and vaccine strategies. Differences were considered significant at a *P* value of <0.05. *, *P* < 0.05; **, *P* < 0.005; ***, *P* < 0.0005; ****, *P* < 0.00005. Solid lines indicate significance within vaccine strategies, and dashed lines indicate significance between lean and obese groups. The dashed red line indicates the limit of detection for the assay.

**FIG 6:**
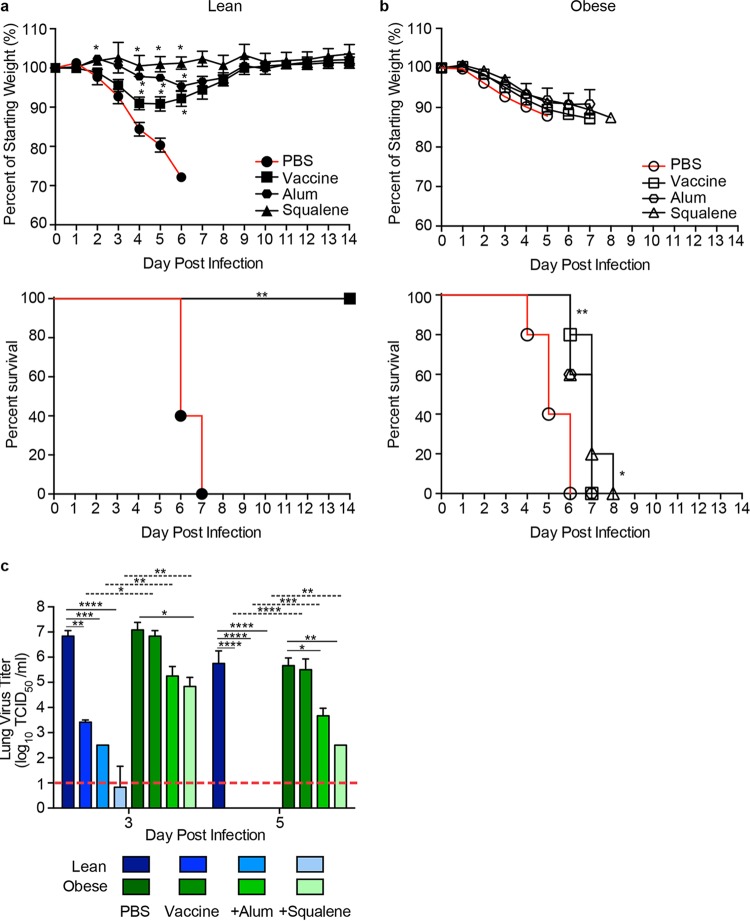
Survival, weight loss, and lung viral titers in vaccinated lean and obese mice following H1N1 virus challenge. (a, b) Three weeks postboost, lean (solid symbols) (a) and obese (open symbols) (b) mice (*n* = 5 or 6/type/group) were challenged with 100× MLD_50_ of influenza virus A/California/04/2009. Mice were monitored for weight and survival daily for 14 days postinfection. Weight data are presented as mean values ± standard errors. Statistical significance was determined using ANOVA, with a *P* value of <0.05 deemed significant compared to the PBS controls. Survival data are presented as the percentages of animals surviving among the total number monitored. Statistical significance was determined by log rank (Mantel-Cox) test, with a *P* value of <0.05 deemed significant compared to the PBS control group. *, *P* < 0.05; **, *P* < 0.005. (c) Viral titers were determined in lungs from vaccinated lean (blue) and obese (green) mice at day 3 and day 5 postinfection with influenza virus A/California/04/2009. Data are presented as the mean log10 TCID_50_/ml ± standard error. Statistical significance was determined using ANOVA, with vaccine strategy, mouse type, and day postinfection as the main effects. Tukey’s test was used for *post hoc* comparison between days postinfection, mouse types, and vaccine strategies. Differences were considered significant at a *P* value of <0.05. *, *P* < 0.05; **, *P* < 0.005; ***, *P* < 0.0005; ****, *P* < 0.00005. Solid lines indicate significance within vaccine strategies, and dashed lines indicate significance between lean and obese groups. The dashed red line indicates the limit of detection for the assay.

Although the titers were lower than in lean mice, obese mice receiving both adjuvanted vaccines did generate neutralizing and nonneutralizing antibody titers that we hypothesized would be protective against influenza virus challenge. To test this, vaccinated lean and obese mice were challenged with 10^5.5^ 50% tissue culture infective dose (TCID_50_) A/Anhui/1/2013 A(H7N9) or 10^4.5^ TCID_50_ pdmH1N1 influenza virus 3 weeks postboost and monitored for morbidity. All lean mice receiving adjuvanted vaccines were protected from influenza virus challenge and had only minimal (5 to 10%) weight loss (Fig. 5A and 6A). In addition, 80% of lean mice receiving unadjuvanted A(H7N9) vaccine were also protected from challenge, although their weight loss was significantly higher (~20%). This could be due to the increased neutralizing (Fig. 1B) and nonneutralizing stalk antibodies (see Fig. S2 in the supplemental material) in vaccinated lean mice. In comparison, all lean mice receiving seasonal A(pdmH1N1) vaccine, regardless of adjuvants, were fully protected from challenge, with good generation of both neutralizing (Fig. 2A and B) and nonneutralizing (see Fig. S2) antibodies.

In contrast, vaccinated obese mice were not protected from challenge irrespective of adjuvant use. For A(H7N9) challenge, all obese mice lost weight and rapidly succumbed to infection in spite of a slight increase in weight at 5 days postinfection (dpi) in the adjuvanted-vaccine group (Fig. 5B). As with previous studies, the failure to protect obese mice is not unique to the A(H7N9) vaccine. As shown by the results in Fig. 6B, we and others (17, 18) demonstrated that seasonal influenza vaccines also fail to protect obese mice.

The reduced protection in vaccinated obese mice was associated with increased viral loads compared to the viral loads in vaccinated lean mice. For both vaccines, at 3 dpi, lean mice receiving the unadjuvanted vaccine had significantly reduced viral loads in the lungs compared to those of unvaccinated controls, with the levels reduced to the limit of detection by 5 dpi (Fig. 5C and 6C). Lean mice receiving adjuvanted vaccine had nearly undetectable viral loads in lung samples at 3 and 5 dpi. In contrast, titers were increased in the obese mice and remained above the limit of detection until the time of death even in mice receiving adjuvanted vaccine (Fig. 5C and 6C), although the adjuvanted vaccine did reduce the viral loads compared to those in the unadjuvanted or unvaccinated mice (Fig. 5C and 6C).

### Increasing the vaccine dose has no impact on survival in obese mice.

**FIG 7:**
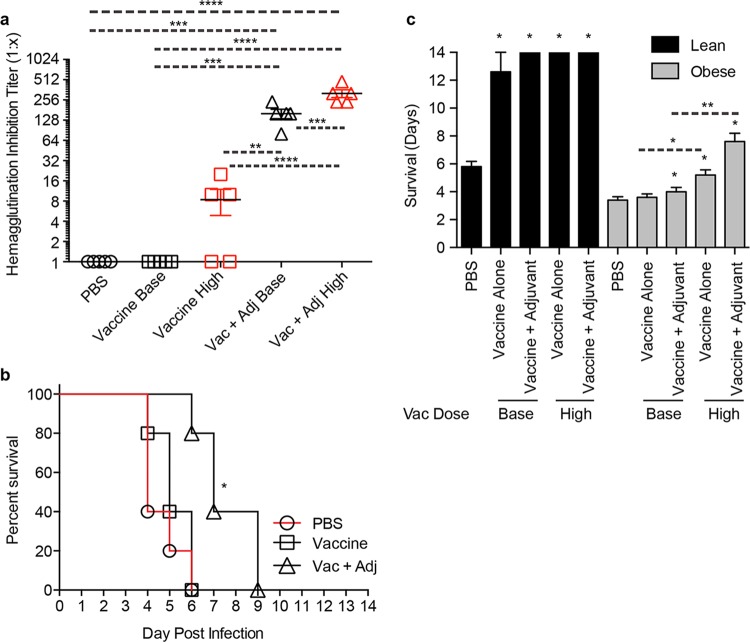
Increasing the vaccine dose does not confer protection in obese mice. (a) Obese mice were vaccinated with a 4× increased dose of vaccine and bled for serological analyses. (b) Mice were then challenged with influenza virus A/Anhui/1/2013 and monitored for survival daily for 14 days postinfection. Statistical significance was determined by log rank (Mantel-Cox) test, with a *P* value of <0.05 deemed significant compared to the PBS control group. *, *P* < 0.05. (c) Days of survival are presented as the number of days mice survived after influenza virus challenge ± standard error. Statistical significance was determined using ANOVA, with vaccine strategy and mouse type as the main effects. A *P* value of <0.05 compared to the PBS control group was deemed significant. *, *P* < 0.05; **, *P* < 0.005; ***, *P* < 0.0005; ****, *P* < 0.00005. Asterisks alone indicate significance compared to the PBS control group, and dashed lines indicate significance between vaccine strategies.

A recent phase 4 clinical trial in the elderly showed that increasing the dose of vaccine antigen significantly increased vaccine effectiveness in this high-risk population (25). Given that obesity induces an immunocompromised state similar to age (16) and that the standard vaccination regimen did not confer protection, we tested whether an increased antigenic dose would protect obese mice from A(H7N9) challenge. Thus, mice were vaccinated with a 4× increased dose of unadjuvanted or adjuvanted vaccine and challenged with influenza virus A/Anhui/1/2013. As expected, all of the vaccinated lean mice were protected from challenge. In contrast, while the increased vaccine dose in conjunction with adjuvant did significantly increase the antibody response compared to the response to the basal dose in the obese mice (Fig. 7A), none of the high-dose-vaccinated obese mice were protected, indicating that an increased vaccine dosage may not increase overall vaccine efficacy in an obese population (Fig. 7B), although it did significantly increase the average number of days of survival compared to the survival of mice receiving the standard vaccine regimen for both the unadjuvanted and adjuvanted groups, with average increases of 1.2 days and 3.6 days, respectively (Fig. 7C). Overall, these studies demonstrate that adjuvanted vaccine failed to protect obese mice from influenza challenge, even when the vaccine antigen dose is increased, and the viral titers remained elevated in spite of the generation of robust antibody responses.

### Passive immunization fails to protect obese mice.

**FIG 8:**
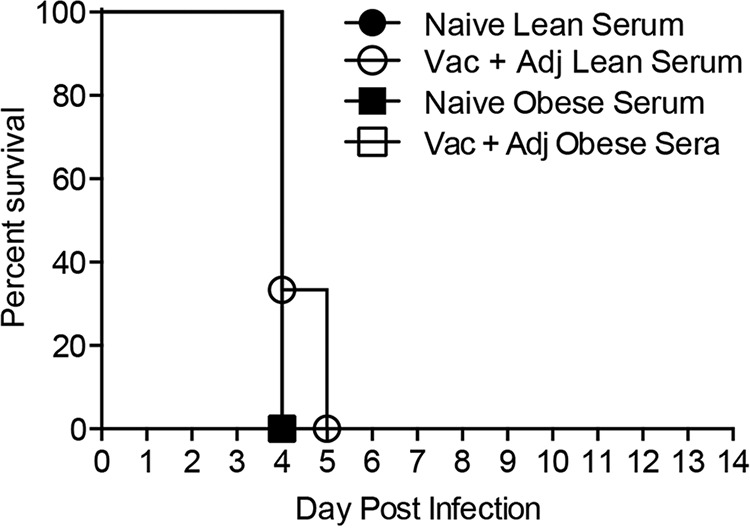
Survival in obese mice following passive serum transfer and H7N9 virus challenge. Sera containing equivalent antibody titers from either naive (solid symbols) or vaccine-plus-adjuvant-vaccinated (open symbols) lean (circles) or obese (squares) mice were passively transferred to 8-week-old obese mice (*n* = 3/group) intraperitoneally, and then the passively immunized mice were challenged with influenza virus A/Anhui/1/2013. Mice were monitored for survival daily for 14 days postinfection. Survival data are presented as the percentages of animals surviving among the total number monitored. Statistical significance was determined by the log rank (Mantel-Cox) test, with a *P* value of <0.05 deemed significant compared to the unvaccinated serum control group. The data shown are representative of two separate experiments.

Although the adjuvanted vaccine did induce antibodies in obese mice, they were not protected from infection. To determine whether this was a problem with the antibodies themselves or the microenvironment, we passively immunized obese mice with postvaccination lean sera. Briefly, naive obese mice were intraperitoneally administered sera containing equivalent IgG amounts from lean and obese mice that were either unvaccinated or had received the AS03-adjuvanted A(H7N9) vaccine and were then challenged with 100× 50% mean lethal dose (MLD_50_) of influenza virus A/Anhui/1/2013 at 24 h posttransfer. The antibody levels pre- and posttransfer were confirmed by both IgG ELISA and HAI (see Fig. S3 in the supplemental material). In contrast to lean mice receiving lean sera, obese mice were not protected from infection regardless of the serum source (Fig. 8). These data demonstrate that the administration of known protective antibodies failed to protect obese mice from influenza virus challenge, which may indicate that the problem lies with the obese host response rather than the protective capacity of antibody responses.

### Obese mice have partial protection at lower viral doses.

**FIG 9:**
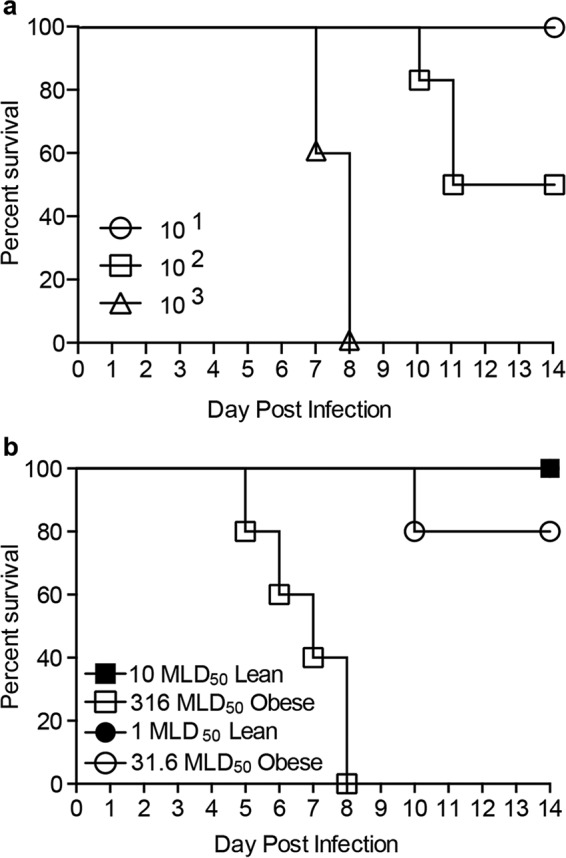
Obese mice are more susceptible to severe influenza virus infection and not fully protected from decreased viral dose. (a) Obese mice were challenged with decreasing amounts of influenza virus A/Anhui/1/2013 (H7N9) to calculate the MLD_50_. (b) Three weeks after being boosted with a standard vaccine, lean (solid symbols) and obese (open symbols) mice (*n* = 5/type/group) were challenged with influenza virus A/Anhui/1/2013 (H7N9) at 10× MLD_50_ for lean mice/316× MLD_50_ for obese mice or 1× MLD_50_/31.6× MLD_50_ for obese mice. Survival was monitored for 14 days postinfection.

Obesity is associated with increased risk of developing severe and even fatal influenza virus infection (1–3, 26). Indeed, we have previously shown that significantly less pandemic H1N1 virus is required to induce disease in obese mice than in lean mice (27, 28). Thus, we questioned whether the extreme susceptibility of obese mice to severe influenza virus infection overrode the protection conferred by vaccination. First we determined the MLD_50_s of the A(H7N9) virus in lean and obese mice. The MLD_50_ in obese mice is 10^2^, compared to 10^3.5^ in lean animals, a 30× decrease (Fig. 9A), suggesting that our 100× LD_50_ challenge dose, which was determined using lean animals, was actually a 3,000× dose in the obese mice. Therefore, mice were administered vaccine plus adjuvant and challenged with 1× and 10× MLD_50_ based on lean mice (31.6 and 316 MLD_50_ for obese mice) 3 weeks postboost (Fig. 9B). As expected, lean mice were completely protected. In contrast, vaccination failed to protect 100% of the obese mice even at the lowest dose, indicating that the hypersusceptibility of the obese host to severe disease could be contributing to the decreased vaccine efficacy. Whether this finding holds true in humans requires further study.

## DISCUSSION

In this study, we demonstrate that the addition of both alum and the squalene oil-in-water adjuvant, ASO3, to an influenza vaccine increased seroconversion in obese mice with two different influenza vaccines. Although lower than the responses in lean mice, obese mice mounted significant neutralizing and nonneutralizing antibody responses toward both vaccines with the addition of either adjuvant. And yet, despite the increased seroconversion, obese mice were not protected from either influenza virus challenge and had significantly increased morbidity and mortality compared to those in the lean controls. These findings highlights that more work may be needed, especially in high-risk populations, to determine the immune correlates of protection, particularly the long-held paradigm that an HAI titer of >40 or a fourfold increase in titer from baseline levels is sufficient for protection against infection (19). Our study suggests that the currently used influenza immune correlate is not suited for the obese population. Coupled with the increasing incidence of obesity in the United States, this highlights the need for identifying novel correlates of protection for influenza vaccination.

In spite of generating neutralizing and nonneutralizing antibodies, obese mice were not protected from influenza virus challenge and had delayed viral clearance compared to that in lean controls. Therefore, the increased morbidity and mortality seen in these mice is likely due to the inability to mitigate viral infection. Increasing the vaccine dose had no impact on morbidity. In addition to the observed differences in the ability to mount a protective antibody response following vaccination, obese mice have additional deficiencies that make them more susceptible to severe influenza virus infections. Previous studies in our laboratory and others (27, 29) have shown that obesity increases the severity of disease from both mouse-adapted and human influenza virus strains. While the exact mechanisms for this increased severity of influenza virus infection in the obese host are unknown, obesity-associated decreases in key immunologic and wound repair functions critical for appropriate response to respiratory infection are implicated (27, 30). Indeed, while obese mice did have a significantly lower MLD_50_ than lean controls, lowering the viral challenge dose below 100× MLD_50_ in the obese animals did not result in complete protection, suggesting that both vaccine response and susceptibility to infection can play a factor in the obese host.

The results from this study raise major concerns over vaccination effectiveness in the obese host. Studies of influenza vaccine responses in obese adults and children have had various results, with some studies showing no differences between lean and obese groups based on serological responses (16, 31–34); however, our studies may indicate that even if the obese population appears serologically protected, effectiveness may be decreased and increased incidence of influenza may occur in an increasingly obese population. Future studies will focus on these questions and try to elucidate why obesity increases the susceptibility to and severity of influenza virus infection, as well as the underlying immunological mechanisms that lead to lack of protection even following adjuvanted vaccination.

## MATERIALS AND METHODS

### Ethics statement.

All procedures were approved by the St. Jude Children’s Research Hospital Institutional Biosafety Committee (IBC) and Animal Care and Use Committee (IACUC) and were in compliance with the Guide for the Care and Use of Laboratory Animals. These guidelines were established by the Institute of Laboratory Animal Resources and approved by the Governing Board of the U.S. National Research Council.

### Laboratory facilities.

All infection experiments were conducted in a biosafety level 3 enhanced containment laboratory (35). Investigators were required to wear appropriate respirator equipment (Racal Health and Safety, Inc., Frederick, MD). Mice were housed in HEPA-filtered, negative-pressure, vented isolation containers.

### Vaccines and adjuvants.

The H7N9 vaccine and AS03 adjuvant were provided by the Office of Biomedical Development Advanced Research and Development Authority. The H7N9 vaccine is a split-virion vaccine derived from A/Shanghai/2/2013 (H7N9), manufactured by Sanofi-Pasteur. AS03 (GlaxoSmithKline) is a squalene-based oil-in-water emulsion containing the immunostimulant α-tocopherol (vitamin E) (36). For vaccination against pdmH1N1, mice were vaccinated with an influenza virus A (pdmH1N1) 2009 monovalent vaccine containing BPL-inactivated, sucrose purified A/California/04/2009. Aluminum hydroxide adjuvant was given as Imject alum adjuvant (Pierce).

### Viruses.

For the pdmH1N1 studies, mice were challenged with A/California/04/2009. For the H7N9 studies, mice were challenged with A/Anhui/1/2013 H7N9 virus, which is antigenically similar to the vaccine strain and causes relatively severe disease in mice (14, 28). All viral stocks were propagated in the allantoic cavity of 10-day-old specific-pathogen-free embryonated chicken eggs at 37°C. Allantoic fluid was harvested, cleared by centrifugation, and stored at −80°C as described previously (37, 38). Viral titers were determined by 50% tissue culture infectious dose (TCID_50_) analysis as previously described (37).

### Cells and culture medium.

MDCK cells were cultured in Eagle’s minimum essential medium (MEM; Mediatech, Manassas, VA) supplemented with 2 mM glutamine and 10% fetal bovine serum (FBS; Gemini Bioproducts, West Sacramento, CA) and grown at 37°C under 5% CO_2_.

### Animal experiments.

Six-week-old C57BL/6 (lean) and B6.Cg-*Lep^ob^*/J (obese) mice (Jackson Laboratory, Bar Harbor, ME) were bled for baseline sera, lightly anesthetized with isoflurane, and vaccinated (*n* = 10 or 11/group) with PBS or 0.375 µg (standard dose) or 1.4 µg (high dose) H7N9 vaccine with or without adjuvant. Three weeks after the initial vaccination, mice were bled and then boosted. Three weeks postboost, animals were lightly anesthetized with isoflurane, bled, and then inoculated intranasally with PBS or 100×, 10×, or 1× (10^5.5^, 10^4.5^, and 10^3.5^ TCID_50_, respectively) MLD_50_ (based on lean animals) of virus in 30 µl PBS. Mice were monitored daily for clinical signs of infection and weighed every 24 h postinfection (39). At days 3 and 5 after 100× MLD_50_ infection, mice (*n* = 3/group) were euthanized, and tissues were harvested and processed immediately or stored at −80°C for future analysis. Moribund mice losing more than 30% body weight and reaching a specified body condition index score were humanely euthanized. For passive transfer experiments, serum was prepared from vaccine-plus-adjuvant-vaccinated mice to equivalent IgG levels, and then 100 µl was injected into lean or obese mice intraperitoneally. Twenty-four hours posttransfer, mice were bled and infected with 100× MLD_50_ of virus as described above.

### Influenza virus-specific antibody determination.

Mouse sera were treated with receptor-destroying enzyme (RDE; Seiken), and hemagglutination inhibition (HAI) assays were performed according to WHO guidelines (40). Luminescent microneutralization (MN) assays were performed as previously described, using a reverse genetics-generated A/Anhui/1/2013 virus or A/California/04/2009 virus containing an NLuc (NanoLuc luciferase) on its polymerase segment (41). Neuraminidase inhibition assays were conducted by enzyme-linked lectin assay for N1- and N9-specific antibodies as previously described (14), using a recombinant reverse genetics (rg)-H6N9 virus with the NA from A/Anhui/1/H7N9 (H7N9) and a mismatched HA from A/Teal/Hong Kong/W312/1997 (H6N1) or an A/California/04/2009 virus.

### Influenza-specific ELISA.

H7N9-specific IgG ELISAs were performed as described previously (42), utilizing horseradish peroxidase-conjugated goat anti-mouse antibodies (Southern Biotech, Birmingham, AL).

### Stalk antibody ELISA.

Recombinant stalk proteins against H1 and H7 were produced (43, 44), and ELISAs were performed as previously described (45).

### Antigen microarrays.

Peptides 20 amino acids in length spanning the HA and NA proteins of the A/Anhui/1/2013 (H7N9) virus were generated with 15-amino-acid overlaps, resulting in the synthesis of 110 HA peptides and 90 NA peptides synthesized at >90% purity (CPC Scientific). A small number of peptides were synthesized at >70% purity, following multiple synthesis and purification attempts. A poly(K) linker was added to each peptide to increase solubility and to improve the binding orientation of peptides to the Hydrogel slides. The peptides were lyophilized in 5-ml tubes and were stored at 20°C. The peptides were resuspended in 100 µl dimethyl sulfoxide (DMSO) and 400 µl ultrapure water to create a working solution of approximately 2 mg/ml. Peptide stocks were diluted 1:2 in protein-printing buffer (phosphate-buffered saline [PBS] with 0.005% Triton X-100) and printed in triplicate on *N*-hydroxysuccinimide ester-derivatized glass slides (H slides; Schott/Nexterion AG) using a QArray2 microarray instrument (Genetix) with contact microarray pins (SMP2.5B; TeleChem). During printing, the relative humidity was maintained at 50 to 60%. Following printing, the slides were left to dry overnight. The arrays were stored at 20°C. The printed grids were outlined with a PAP hydrophobic pen (Research Products International). The slides were chemically blocked using 4 ml of 50 mM borate, 50 mM ethanolamine for 1 h. The slides were then washed twice with PBS containing 0.05% Tween 20, twice with PBS, and once in deionized water and then spun to dry at 1,000 × *g* for 5 min at room temperature. Serum samples were diluted 1:25 in 1% bovine serum albumin and 0.025% Tween 20, incubated on slides for 2 h in a humidified chamber at 25°C, and then washed twice with PBS containing 0.05% Tween 20 and twice with PBS. Bound immunoglobulins were detected for 45 min with Alexa Fluor 647 goat anti-mouse IgG (115-605-008; Jackson ImmunoResearch). The arrays were washed as noted above and were spun dry as described above. The slides were scanned on a two-laser GenePix 4400SL scanner (Molecular Devices) probing for IgG responses. Images were analyzed using GenePix version 7.2 to obtain the mean fluorescence intensity (MFI) for each probe. All samples were run the same day and processed together. Negative controls were run in triplicate for subtraction of background. Responses below 1,000 MFI after subtraction of background were considered negative (MFI range, 0 to 65,000). Subsequently, all data were analyzed with MatLab (MathWorks) and Python. For each probe, we used the median response and subtracted the average background of multiple negative controls.

### Data representation.

We denote the normalized array measurements by $x_{i,p,a_{p}}$, where *i* is subject, *i* = 1, …, *N*; *p* is pathogen, *p* = 1, …, *P*; *a_p_* is antigen *a* from pathogen *p*, *a_p_* = 1, …, *N_p_*. *z_i_* denotes the treatment assignment (vaccine/placebo) of subject *i* and *y_i_* denotes the outcome of subject *i* (vaccine-induced antibody titer/infection status/disease status); the observed data for each subject are $\left(z_{i},y_{i},x_{i,p,a_{p}}\right)$ for *i* = 1, …, *N*, *p* = 1, …, *P*, and *a_p_* = 1, …, *N_p_*.

The antibody profile generated by the AMs is a multidimensional measurement of the antibody responses to a large set of overlapping peptides from HA and NA. To compare the peptide responses to the HAI and MN assays as measured by the AM for a given subject, we define the breadth *b* and magnitude *m* of responses to each protein as follows:

(1) $$m_{i,p}=\sum_{a_{p}=1}^{N_{P}}x_{i,p,a_{p}}$$

denotes the magnitude of responses to all antigens of pathogen *p* and

(2) $$b_{i,p}=\sum_{a_{p}=1}^{N_{P}}I(x_{i,p,a_{p}}>0)$$

denotes the breadth of response to antigens from pathogen *p*, where *I* denotes the indicator function, and where positivity ($x_{i,p,a_{p}}>0$) is determined using the responses of negative controls.

### Statistical analysis.

For the survival data, statistical significance was determined by log rank (Mantel-Cox) test, with a *P* value of <0.05 deemed significant compared to the data for the PBS control group. For comparisons between groups for the serological and viral studies, statistical significance was determined using analysis of variance (ANOVA), with vaccine strategy, mouse type, and day postinfection as the main effects. Tukey’s test was used for *post hoc* comparison between days postinfection, mouse types, and vaccine strategies. Differences were considered significant at a *P* value of <0.05. For antibody arrays, the responses of groups were compared using the Wilcoxon rank sum test. The responses of each group were summarized by median values. All *P* values for associations between the AM data and HAI and MN assays were computed using Spearman’s rank-order correlation. Adjustments for multiplicity were computed using the Bonferroni correction and were adjusted separately for comparing different vaccine treatments (PBS, vaccine, and vaccine plus adjuvant) within each mouse group and for comparing the same vaccine across the two mouse groups.’s

## SUPPLEMENTAL MATERIAL

Figure S1  Experimental setup. Six-week-old C57BL/6 (lean) and B6.Cg-*Lep^ob^*/J (obese) mice were bled for baseline sera, lightly anesthetized with isoflurane, and then vaccinated (*n* = 10 or 11/group) with PBS or H7N9 vaccine with or without adjuvant. Three weeks after the initial vaccination, the mice were bled and then boosted with a second dose of vaccine or vaccine plus adjuvant. Three weeks postboost, the animals were lightly anesthetized with isoflurane, bled, and then inoculated intranasally with PBS or 100× MLD_50_ of A/Anhui/1/2013 (H7N9) or A/California/04/2009 (H1N1). The mice were monitored daily for clinical signs of infection and weighed every 24 h postinfection. Download Figure S1, PDF file, 0.4 MB

Figure S2  Stalk antibody response following vaccination with unadjuvanted vaccine. Groups of lean (solid symbols) or obese (open symbols) mice (*n* = 10 or 11/type/group) were vaccinated with PBS (circles) or unadjuvanted vaccine (squares). Three weeks postvaccination, mice were boosted, and serum was collected 3 weeks postboost. Postboost serum was analyzed for stalk antibody against influenza virus A/Anhui/1/2013 (H7N9) (a) and influenza virus A/California/04/2009 (pdmH1N1) (b). Data are presented as mean absorbance values ± standard errors. Statistical significance was determined using ANOVA, with vaccine strategy and mouse type as the main effects. Tukey’s test was used for *post hoc* comparison. Differences were considered significant at a *P* value of <0.05. *, *P* < 0.05. Download Figure S2, PDF file, 0.4 MB

Figure S3  Serology in obese mice before and after passive serum transfer. Eight-week-old obese mice (*n* = 3/group) were given a passive transfer of either naive or vaccine-plus-adjuvant-vaccinated lean or obese mouse serum intraperitoneally. Serum IgG (a) and hemagglutination inhibition (b) titers were measured before (black) and after (white) transfer. Download Figure S3, PDF file, 0.4 MB
