# Supplementary material for: Obesity Outweighs Protection Conferred by Adjuvanted Influenza Vaccination
Source: mBio. 2016 Aug 2;7(4):e01144-16. doi: 10.1128/mBio.01144-16 (PMC4981723; doi:10.1128/mBio.01144-16)
Supplement: Figure S1 — Experimental setup. Six-week-old C57BL/6 (lean) and B6.Cg-Lepob/J (obese) mice were bled for baseline sera, lightly anesthetized with isoflurane, and then vaccinated (n = 10 or 11/group) with PBS or H7N9 vaccine with or without adjuvant. Three weeks after the initial vaccination, the mice were bled and then boosted with a second dose of vaccine or vaccine plus adjuvant. Three weeks postboost, the animals were lightly anesthetized with isoflurane, bled, and then inoculated intranasally with PBS or 100× MLD50 of A/Anhui/1/2013 (H7N9) or A/California/04/2009 (H1N1). The mice were monitored daily for clinical signs of infection and weighed every 24 h postinfection. Download [file mbo004162929sf1.pdf]

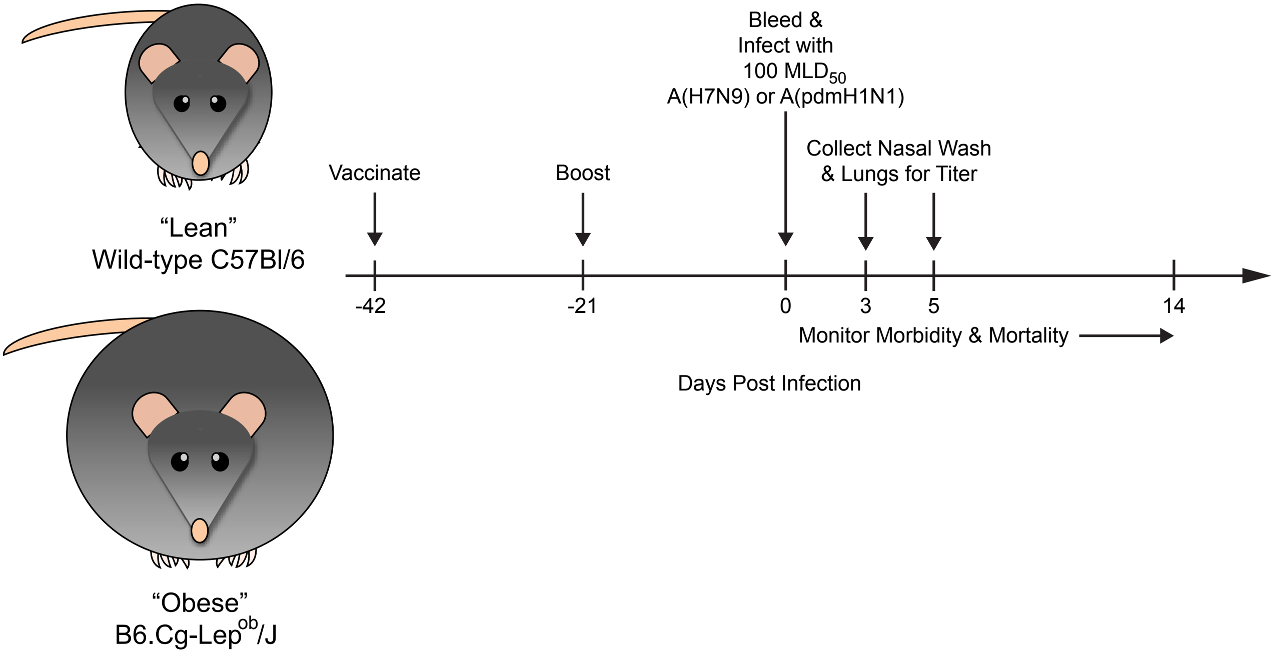

Supplementary Figure S1: Experimental setup. Six week-old C57Bl/6 (lean) and B6.Cg-Lepob/J (obese) mice were bled for baseline sera, lightly anesthetized with isofluorane and then vaccinated (n=10-11/group) with PBS, or H7N9 vaccine with or without adjuvant. Three weeks following initial vaccination, mice were bled then boosted with a second dose of vaccine or vaccine plus adjuvant. Three weeks post boost, animals were lightly anesthetized with isofluorane, bled then inoculated intranasally with PBS or 100x MLD50 of A/Anhui/1/2013 (H7N9). Mice were monitored daily for clinical signs of infection and weighed every 24 hpi.

Supplemental Figure S1
