# Supplementary material for: Obesity Outweighs Protection Conferred by Adjuvanted Influenza Vaccination
Source: mBio. 2016 Aug 2;7(4):e01144-16. doi: 10.1128/mBio.01144-16 (PMC4981723; doi:10.1128/mBio.01144-16)
Supplement: Figure S2 — Stalk antibody response following vaccination with unadjuvanted vaccine. Groups of lean (solid symbols) or obese (open symbols) mice (n = 10 or 11/type/group) were vaccinated with PBS (circles) or unadjuvanted vaccine (squares). Three weeks postvaccination, mice were boosted, and serum was collected 3 weeks postboost. Postboost serum was analyzed for stalk antibody against influenza virus A/Anhui/1/2013 (H7N9) (a) and influenza virus A/California/04/2009 (pdmH1N1) (b). Data are presented as mean absorbance values ± standard errors. Statistical significance was determined using ANOVA, with vaccine strategy and mouse type as the main effects. Tukey’s test was used for post hoc comparison. Differences were considered significant at a P value of <0.05. *, P < 0.05. Download [file mbo004162929sf2.pdf]

a

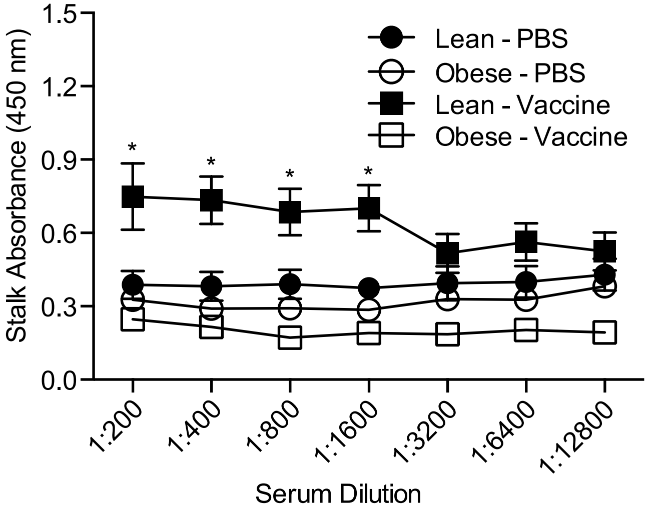

b

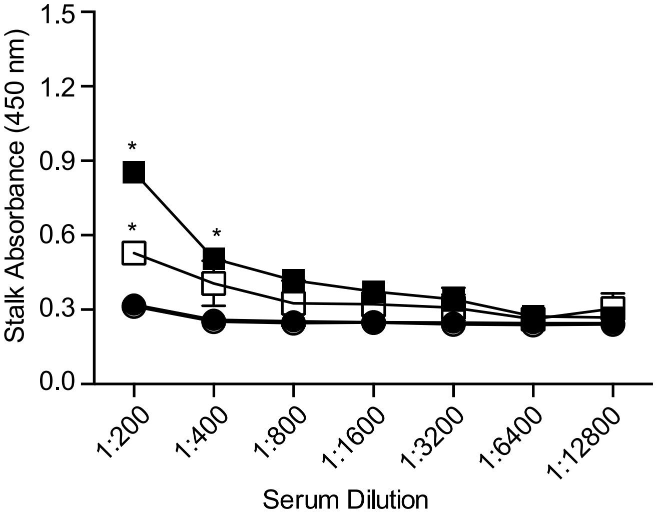

Supplementary Figure S2: Stalk antibody response following vaccination alone. Groups of lean (solid) or obese (open) mice (n = 10-11/type/group) were vaccinated with PBS (circles), vaccine alone (squares). Three weeks post vaccination, mice were boosted and serum was collected 3 weeks post boost. Post-boost serum was analyzed for stalk antibody against influenza A/Anhui/1/2013 (H7N9) (A) and influenza A/California/04/2009 (pdmH1N1) (B). Data are presented as mean absorbance ± standard error. Statistical significance was determined using ANOVA with vaccine strategy and mouse type as main effects. Tukey's was used for post-hoc comparison. Differences were considered significant at $p<0.05$. *$p<0.05$.

Supplemental Figure S2
