# Supplementary material for: Obesity Outweighs Protection Conferred by Adjuvanted Influenza Vaccination
Source: mBio. 2016 Aug 2;7(4):e01144-16. doi: 10.1128/mBio.01144-16 (PMC4981723; doi:10.1128/mBio.01144-16)
Supplement: Figure S3 — Serology in obese mice before and after passive serum transfer. Eight-week-old obese mice (n = 3/group) were given a passive transfer of either naive or vaccine-plus-adjuvant-vaccinated lean or obese mouse serum intraperitoneally. Serum IgG (a) and hemagglutination inhibition (b) titers were measured before (black) and after (white) transfer. Download [file mbo004162929sf3.pdf]

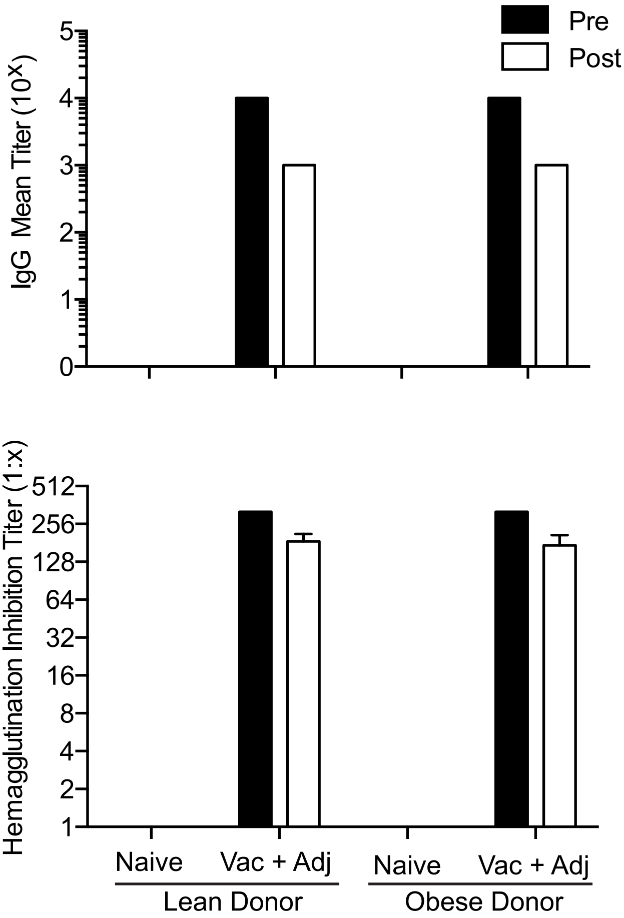

Supplemental Figure S3: Serology in obese mice pre and post-passive serum transfer. 8 week old, obese mice (n = 3/group) were given a passive transfer of either naïve or adjuvanted vaccine lean or obese sera intraperitoneally. Serum IgG (A) and hemagglutination inhibiton (B) titers were measured before (black) and following (white) transfer.

Supplemental Figure S3
